# Draft genome sequence of *Clostridium thermobutyricum* from the swine gut

**DOI:** 10.1128/mra.00724-25

**Published:** 2026-04-01

**Authors:** Jinling He, Zhijian Xu, Hui Jiang, Jianmin Chai, Xiaofan Wang, Feilong Deng, Jiangchao Zhao, Ying Li

**Affiliations:** 1 Guangdong Provincial Key Laboratory of Animal Molecular Design and Precise Breeding, School of Animal Science and Technology, Foshan University 47868, Foshan, China; 2 School of Animal Science and Technology, Foshan University 47868, Foshan, China; 3 College of Animal Science, South China Agricultural University 527517, Guangzhou, China; Fluxus Inc., Sunnyvale, California, USA

**Keywords:** whole-genome sequencing, fecal microbiota, swine, *Clostridium thermobutyricum*, butyrate

## Abstract

We report the isolation and draft genome sequence of *Clostridium thermobutyricum* from swine feces. The assembly comprises 82 scaffolds, totaling 3,236,413 bp with a GC content of 26.47%, and shows 99.19% estimated completeness and 0.81% contamination.

## ANNOUNCEMENT

Recent research has highlighted the important influence of microorganisms on their hosts (1). *Clostridium thermobutyricum* is a thermophilic butyrate-producing bacterium distinct from *C. butyricum* in taxonomy, physiology, and ecological roles (2). Its ability to thrive at 50°C–60°C and utilize thermostable metabolic pathways highlights potential applications in high-temperature fermentation and bioenergy production (3). Despite its relevance, this bacterium remains poorly characterized in the porcine gut. Genome sequencing will elucidate its functional properties and biotechnological potential. High-fidelity metagenomic sequencing has become a widely adopted approach for studying microorganisms (4).

The animal experiments were approved by the Laboratory Animal Ethics Committee of *Foshan University* (Approval No: FOSU20200504). *Clostridium thermobutyricum was* isolated from fecal samples of Small-ear Spotted swine (a Chinese indigenous breed) collected in Shanshui, Foshan City (approximate: 23.0°N, 113.0°E), Guangdong Province, China. The outer exposed layer of the feces was first removed with a cotton swab, followed by collection of the inner fresh fecal material as the sample, which was then transported to the laboratory. The sample was serially diluted 1:10 with 1× PBS and plated onto Reinforced Clostridial Medium (RCM) agar plates. The plates were incubated in a Whitley M35 variable atmosphere workstation under anaerobic conditions (37°C, 8–10 h), after which a single colony was transferred to 1 mL of RCM liquid medium for expansion under the same anaerobic conditions (37°C, 8–10 h) prior to DNA extraction. Genomic DNA was extracted using the TIANcombi DNA Lyse & Det PCR Kit following the manufacturer’s protocol (5). A total of 0.2 μg of genomic DNA was sheared using a Covaris M220 Focused-ultrasonicator (Covaris, USA). DNA quantity and purity were assessed using a NanoDrop spectrophotometer. Precise quantification was performed with a Qubit Fluorometer. DNA integrity was verified using an Agilent 4200 TapeStation prior to library construction. Sequencing libraries were then constructed using the NEBNext Ultra II DNA Library Prep Kit (NEB, USA) according to the manufacturer’s protocol. The libraries were subjected to PE150 sequencing (2 × 150 bp) on the Illumina NovaSeq 6000 system at Novogene Co., Ltd. (Beijing, China), which yielded a total of 6,894,808 reads. After assessing library integrity and insert size distribution with an AATI Fragment Analyzer, libraries were pooled based on their effective concentrations and the required data output, followed by paired-end sequencing. The raw reads were quality-controlled using Trimmomatic (version 0.39-2; key parameters: illumina clip: truseq3-pe.fa:2:30:10, leading:20, trailing:20, slidingwindow:4:25 minlen:100) to generate clean data (6). The genome was assembled from the clean data using SPAdes (version 4.0.0; key parameters: --isolate; --cov-cutoff auto) (7). The quality of the assembled genome was assessed via CheckM (version 1.2.3; key parameters: lineage-wf mode) (8). The assembled genome was annotated using the NCBI Prokaryotic Genome Annotation Pipeline (PGAP, version 6.10) (9). For species-level annotation and classification, GTDB-Tk (version 2.4.0; key parameters: classify-wf mode) was employed (10). Finally, Prodigal (version 6.8; -g 11 -p single) was used to predict functional genes within the genome (11) (Table 1).

**TABLE 1:** Draft genome metrics of *Clostridium thermobutyricum*

Metric	Result	Tool
Number of scaffolds	82	CheckM
Number of contigs	84	CheckM
GC content	26.5%	CheckM
Depth	12×	BWA + samtools
N50	131,925 bp	CheckM
Estimated completeness	99.19%	CheckM
Estimated contamination	0.81%	CheckM
Predicted genes	3,094	Prodigal
Total size	3,236,413 bp	CheckM

*Clostridium thermobutyricum* was isolated for the first time from the pig gut microbiota, and its draft genome was assembled. This discovery provides new insights into the functional potential of this genus in the pig gut.

## Data Availability

The sequencing data generated in this study have been deposited in the NCBI Sequence Read Archive (SRA) under BioProject accession number PRJNA1304223, which is associated with the strain *Clostridium thermobutyricum* S1-18-1 (https://www.ncbi.nlm.nih.gov/sra/?term=SRR35012508). The genome assembly is accessible under the accession number GCF_051362265.1.
